# Spectrum of mutational signatures in T-cell lymphoma reveals a key role for UV radiation in cutaneous T-cell lymphoma

**DOI:** 10.1038/s41598-021-83352-4

**Published:** 2021-02-17

**Authors:** Christine L. Jones, Andrea Degasperi, Vieri Grandi, Tauanne D. Amarante, Genomics England Research Consortium, John C. Ambrose, Prabhu Arumugam, Emma L. Baple, Marta Bleda, Freya Boardman-Pretty, Jeanne M. Boissiere, Christopher R. Boustred, Helen Brittain, Mark J. Caulfield, Georgia C. Chan, Clare E. H. Craig, Louise C. Daugherty, Anna de Burca, Andrew Devereau, Greg Elgar, Rebecca E. Foulger, Tom Fowler, Pedro Furió-Tarí, Adam Giess, Joanne M. Hackett, Dina Halai, Angela Hamblin, Shirley Henderson, James E. Holman, Tim J. P. Hubbard, Kristina Ibáñez, Rob Jackson, Louise J. Jones, Dalia Kasperaviciute, Melis Kayikci, Athanasios Kousathanas, Lea Lahnstein, Kay Lawson, Sarah E. A. Leigh, Ivonne U. S. Leong, Javier F. Lopez, Fiona Maleady-Crowe, Joanne Mason, Ellen M. McDonagh, Loukas Moutsianas, Michael Mueller, Nirupa Murugaesu, Anna C. Need, Peter O’Donovan, Chris A. Odhams, Andrea Orioli, Christine Patch, Mariana Buongermino Pereira, Daniel Perez-Gil, Dimitris Polychronopoulos, John Pullinger, Tahrima Rahim, Augusto Rendon, Pablo Riesgo-Ferreiro, Tim Rogers, Mina Ryten, Kevin Savage, Kushmita Sawant, Richard H. Scott, Afshan Siddiq, Alexander Sieghart, Damian Smedley, Katherine R. Smith, Samuel C. Smith, Alona Sosinsky, William Spooner, Helen E. Stevens, Alexander Stuckey, Razvan Sultana, Mélanie Tanguy, Ellen R. A. Thomas, Simon R. Thompson, Carolyn Tregidgo, Arianna Tucci, Emma Walsh, Sarah A. Watters, Matthew J. Welland, Eleanor Williams, Katarzyna Witkowska, Suzanne M. Wood, Magdalena Zarowiecki, Tracey J. Mitchell, Serena Nik-Zainal, Sean J. Whittaker

**Affiliations:** 1 St. John’s Institute of Dermatology, School of Basic and Medical Biosciences, King’s College London, Guy’s Hospital, London, SE1 9RT UK; 2 MRC Cancer Unit, Hutchison/MRC Research Centre, University of Cambridge, Box 197, Cambridge Biomedical Campus, Cambridge, CB2 0XZ UK; 3 Academic Laboratory of Medical Genetics, Lv 6 Addenbrooke’s Treatment Centre, Addenbrooke’s Hospital, Box 238, Cambridge, CB2 0QQ UK; 4 Genomics England, London, UK; 5 William Harvey Research Institute, Queen Mary University of London, London, EC1M 6BQ UK

**Keywords:** T-cell lymphoma, Cancer genomics

## Abstract

T-cell non-Hodgkin’s lymphomas develop following transformation of tissue resident T-cells. We performed a meta-analysis of whole exome sequencing data from 403 patients with eight subtypes of T-cell non-Hodgkin’s lymphoma to identify mutational signatures and associated recurrent gene mutations. Signature 1, indicative of age-related deamination, was prevalent across all T-cell lymphomas, reflecting the derivation of these malignancies from memory T-cells. Adult T-cell leukemia-lymphoma was specifically associated with signature 17, which was found to correlate with the IRF4 K59R mutation that is exclusive to Adult T-cell leukemia-lymphoma. Signature 7, implicating UV exposure was uniquely identified in cutaneous T-cell lymphoma (CTCL), contributing 52% of the mutational burden in mycosis fungoides and 23% in Sezary syndrome. Importantly this UV signature was observed in CD4 + T-cells isolated from the blood of Sezary syndrome patients suggesting extensive re-circulation of these T-cells through skin and blood. Analysis of non-Hodgkin’s T-cell lymphoma cases submitted to the national 100,000 WGS project confirmed that signature 7 was only identified in CTCL strongly implicating UV radiation in the pathogenesis of cutaneous T-cell lymphoma.

## Introduction

Non-Hodgkin’s lymphomas (NHL) consist of distinct clinico-pathologic and molecular entities arising from nodal or extra-nodal sites^1^. T-cell lymphomas often develop in extra-nodal sites, constitute only 10–15% of NHL in the west, although higher rates of 20–25% are reported in Asia, and arise from tissue resident αβ or γδ T-cells^2^. Whilst the incidence of NHL has increased, the underlying aetiology remains poorly understood.

Recurrent chromosomal translocations are common in B-cell NHL and are due to intrinsic molecular mechanisms including aberrant V(D)J recombination in which different genes are juxtaposed with immunoglobulin promotors, somatic hypermutation and class switch recombination^3^. For mature T-cell NHL, the prevalence of recurrent translocations is less, with notable exceptions such as the t(2;5) in ALK + anaplastic large cell lymphomas^4^, reflecting the lack of somatic hypermutation and class switch recombination in healthy mature T-cells. In contrast the identification of extrinsic factors has proved elusive, with the exception of viral transformation such as Adult T-cell leukemia-lymphoma (ATLL) associated with HTLV-1^5^ and EBV associated NHL^6^.

A combination of intrinsic and extrinsic mutational processes cause a characteristic mutational spectrum. Consequently when a cell is clonally expanded through malignancy, these patterns can be detected within the tumour. The patterns of mutation are defined by assessing the six substitution types which can occur (C>A, C>G, C>T, T>A, T>C, and T>G) and considering the 5′ and 3′ nucleotide context giving 96 different trinucleotide mutation types^7^. An analysis of over 10,000 exomes and 1000 genomes across 40 types of cancer in The Cancer Genome Atlas (TCGA) identified 30 distinct signatures, some of which can be attributed to known mutagens (COSMIC mutational signatures v2, available at https://cancer.sanger.ac.uk/cosmic/signatures_v2).

Previous studies showed that mutational signatures in B-cell NHL including B-CLL are often associated with intrinsic abnormalities such as the COSMIC signature 9 due to somatic hypermutation associated with aberrant activation induced cytosine deaminase^8^. Furthermore, single cell whole genome sequencing studies of B-cells from healthy volunteers suggests that mutational signatures correlate with intrinsic factors including the age associated COSMIC signature 1 due to spontaneous deamination of methylated cytosines as well as COSMIC signature 9^9^. As T-cell lymphomas are not included in TCGA, there have been no comprehensive studies of mutational signatures in T-cell NHL although recent high-throughput sequencing studies have identified putative driver gene mutations targeting specific signalling pathways notably TCR, NF-kB and JAK-STAT signalling^10–14^. These findings suggest that some T-cell NHL are dependent on TCR signalling analogous to a dependence on BCR signalling in B-cell NHL^15^.

In order to identify causal factors in T-cell NHL, we performed a systematic review of the literature and identified 14 published WES studies of T-cell NHLs from which mutational data could be combined^11–14,16–25^. Using this dataset of over 400 whole exome sequences we deconvoluted the mutational signatures present in eight different subtypes of mature T-cell lymphomas. Whilst this analysis indicates that all mature T-cell NHLs have age related signatures, distinct signatures are present, including signature 17 associated with HTLV-1 transformed ATLL and UV signature 7 in cutaneous T-cell lymphoma (CTCL). We show that this clonotypic UV signature is present in leukemic T-cells, presumably due to recirculation from skin. These findings have important implications for the pathogenesis of CTCL and suggest that skin resident memory T-cells are susceptible to the mutagenic as well as the immunomodulatory effects of UV.

## Results

### T-cell lymphomas have a variable mutational load

Since mature T-cell NHLs have not been included in any of the large pan-cancer sequencing projects, we performed a comprehensive review of the literature. 34 studies were identified which included whole exome data from 631 patients. Overall 14 studies comprising 403 patients met our eligibility criteria (Table 1).

**Table 1:** Details of the studies included and the number of samples with each disease subtype.

	AITL	ATLL	EATL	HSTL	MF	NKTCL	PTCL	SS	
Choi 2015^16^	–	–	–	–	–	–	–	33	33
da Silva Almeida 2015^17^	–	–	–	–	8	–	–	26	34
Jiang 2015^12^	–	–	–	–	–	25	–	–	25
Kataoka 2015^11^	–	81	–	–	–	–	–	–	81
McKinney 2017^13^	–	–	–	64	–	–	–	–	64
Moffitt 2017^14^	–	–	58	–	–	–	–	–	58
Palomero 2014^18^	3	–	1	–	–	2	6	–	12
Prasad 2016^19^	–	–	–	–	–	–	–	12	12
Roberti 2016^20^	–	–	15	–	–	–	–	–	15
Sakata–Yanagimoto 2014^21^	3	–	–	–	–	–	3	–	6
Ungewickell 2015^22^	–	–	–	–	6	–	–	5	11
Wang 2015^23^	–	–	–	–	–	–	–	37	37
Woollard 2016^24^	–	–	–	–	–	–	–	10	10
Yoo 2014^25^	5	–	–	–	–	–	–	–	5
	11	81	74	64	14	27	9	123	

**Figure 1:**
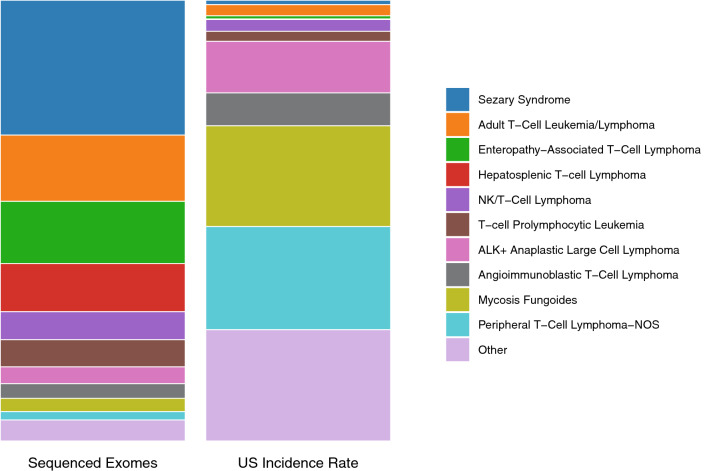
Comparison of the distribution of number of exomes sequenced with the US incidence rate of each subtype. Literature analysis identified 631 whole exome sequences performed on mature T-cell NHL samples. US Incidence rates are derived from the 2016 US lymphoid malignancy statistics^26^ which estimated 8,380 new cases of mature T-cell NHL in 2016.

There was a striking disparity in the number of exomes sequenced between the subtypes, particularly when compared to the relative incidence of each subtype (Fig. 1). Notably, rare subtypes such as Sezary syndrome (SS) and ATLL account for 45% of the exomes sequenced whilst only representing 4% of mature T-cell NHL cases in the US^26^. On the other hand, mycosis fungoides (MF) and peripheral T-cell lymphoma (PTCL) account for only 5% of the exomes sequenced whilst representing 45% of mature T-cell NHL cases. This is likely because both SS and ATLL are leukaemic variants facilitating isolation of clonal populations of tumour cells from peripheral blood.

**Figure 2:**
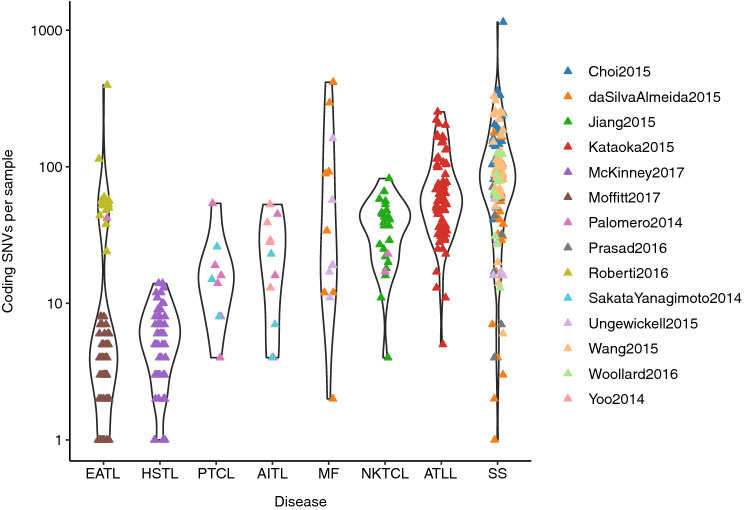
Number of coding SNVs per sample across disease subtypes. Data were collated for those studies where full mutation lists were published and for subtypes where sequencing had been performed on at least 10 patients.

Examining the distribution of mutational burden within and between subtypes of T-cell lymphoma revealed that SS has both the highest median number of coding SNVs per sample and the widest range (Fig. 2), supporting previous observations of heterogeneity in SS^10^. ATLL, which is driven by HTLV-1, has a high mutation rate with a much narrower distribution. Enteropathy-associated T-cell lymphoma (EATL) and hepatosplenic T-cell lymphoma (HSTL), which often arise from γδ T-cells, have the lowest mutation rate, although it is notable that the EATL samples have a bimodal distribution. This split corresponds to the study from Roberti et al.^20^ averaging 77 SNVs and Moffitt et al.^14^ averaging 2.5 per sample. The one sample from Palomero et al.^18^ sits with the higher group at 42 suggesting that the filtering criteria used by Moffitt *et. al.* may have been more stringent. This highlights that, because our analysis was based on published data from multiple sources, differences in sequencing depth and filtering strategy could contribute to variability in results for other subtypes as well. However, samples from multiple SS studies were analysed and showed comparable mutational burden suggesting that technical differences did not contribute significantly to variation in SS results.

### Recurrent driver mutations across T-cell NHL subtypes

**Figure 3:**
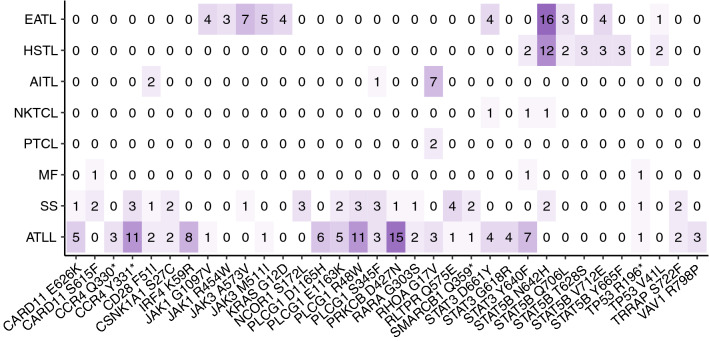
Recurrent driver gene mutations in mature T-cell NHL. Heatmap showing the number of patients with a given driver gene mutation for each subtype where a given variant occurred in three or more patients.

Recurrent mutations in the driver genes, as defined by Park *et. al.*^10^ were assessed in three or more patients within the cohort and their distribution compared across disease subtypes (Fig. 3). Overall 34 recurrent mutations were identified in 20 genes across different T-cell lymphoma subtypes. EATL, HSTL, Angioimmunoblastic T-cell lymphoma (AITL) and ATLL each have at least one highly recurrent mutation, while the other subtypes show a more heterogeneous pattern. 31 of the 34 recurrent mutations are listed in the COSMIC catalogue, of which 90% are associated with haematologic malignancies^27^. In addition, functional characterisation has predominately shown gain of function mutations leading to increased TCR signalling and proliferation. Key exceptions are RHOA G17V, a loss of function mutation leading to dominant negative inhibition of wild type RHOA GTPase activity^18,21^ and TP53 R196* a truncation which lacks tumour suppressor activity and is observed in a wide range of solid tumours^28^.

Recurrent JAK mutations were mostly restricted to EATL whilst recurrent STAT mutations were prevalent in both EATL and HSTL. Since EATL and HSTL frequently involve γδ T-cells, we checked whether the recurrent JAK/STAT mutations were restricted to T-cell lymphomas derived from γδ as opposed to αβ T-cells. However no association was found suggesting that the restriction of certain recurrent mutations to particular disease subtypes reflects the tissue specificity of those T-cells rather than the TCR type. Several recurrent mutations involved different nucleotide changes resulting in the same amino acid change. For example JAK3 M511I was caused by a C>A substitution in 3 patients and a C>G substitution in 3 patients indicating a selection pressure for the same amino acid change even though it may be generated through different mutational processes.

### Landscape of Mutational Signatures in T-cell NHL

**Figure 4:**
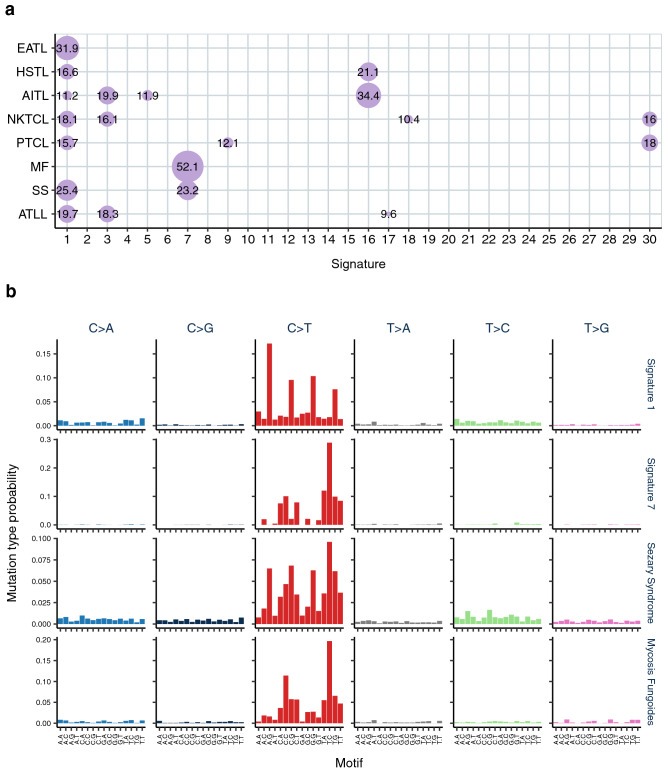
Mutational signatures identified in T-cell lymphoma. (**a**) matrix showing the percentage of mutations in each disease subtype assigned to the 30 COSMIC signatures. (**b**) plots showing the likelihood of each mutation in each trinucleotide context for Signatures 1 and 7 as per COSMIC mutational signatures v2 and the observed SNV catalogues for Sezary syndrome and mycosis fungoides.

The full list of exome SNVs were grouped by disease subtype and subject to signature fitting against the 30 COSMIC signatures. This revealed contributions from nine signatures across all the subtypes with signature 1, the age-related deamination of 5-methylcytosine, being the most prevalent (Fig. 4a). Comparison between the original mutational catalogues and reconstructed profiles based on the extracted signature contributions suggested that no further undefined signatures were involved. A lower cosine similarity was observed between original and reconstructed plots for PTCL and HSTL but this probably reflects a lower total number of SNVs compared to the other subtypes. In the context of exome sequencing data, signatures such as 3, 5, and 8 are challenging to assign due to their relatively flat and featureless SNV pattern and may well be false positives^29^. Hence, we focussed on signatures with a distinctive profile and potential functional relevance.

Cutaneous T-cell lymphomas, MF and SS, showed a strong contribution from signature 7 (Fig. 4b) indicating a potential causal link with UV exposure. Data were also examined on a study specific level and all eight studies of CTCL patients demonstrated presence of the UV signature contributing between 10.8 and 57.6% of the mutational burden. Crucially, all the SS samples were obtained from peripheral blood with a dominant clonal T-cell population and 41% of patients were treatment naïve at the time of sampling. This suggests that the leukaemic T-cells in SS must have been circulating within the skin for a prolonged period prior to initiation of clonal expansion in order to accumulate a large number of UV mutations.

To establish whether all SS patients showed a contribution from signature 7, we analysed exome SNVs from individual SS patients. Of the 90 SS patients with sufficient mutations to define signatures, 67 (74%) had a signature 7 contribution representing between 7.5 and 88% of the overall SNV catalogue (Supplementary Table 1). However there was no correlation between the percentage contribution of signature 7 and mutational burden in SS patients. Overall 41% of SS patients were treatment naïve at the time of analysis and, where it was possible to identify treatment of individual patients, 78% of treatment naïve patients showed a signature 7 contribution. Of those who were treated, 27 patients received photopheresis whilst 13 received phototherapy (11 UVB and 2 PUVA), however this information was provided in summary form and so it was not possible to allocate treatments to samples from specific individuals.

**Figure 5:**
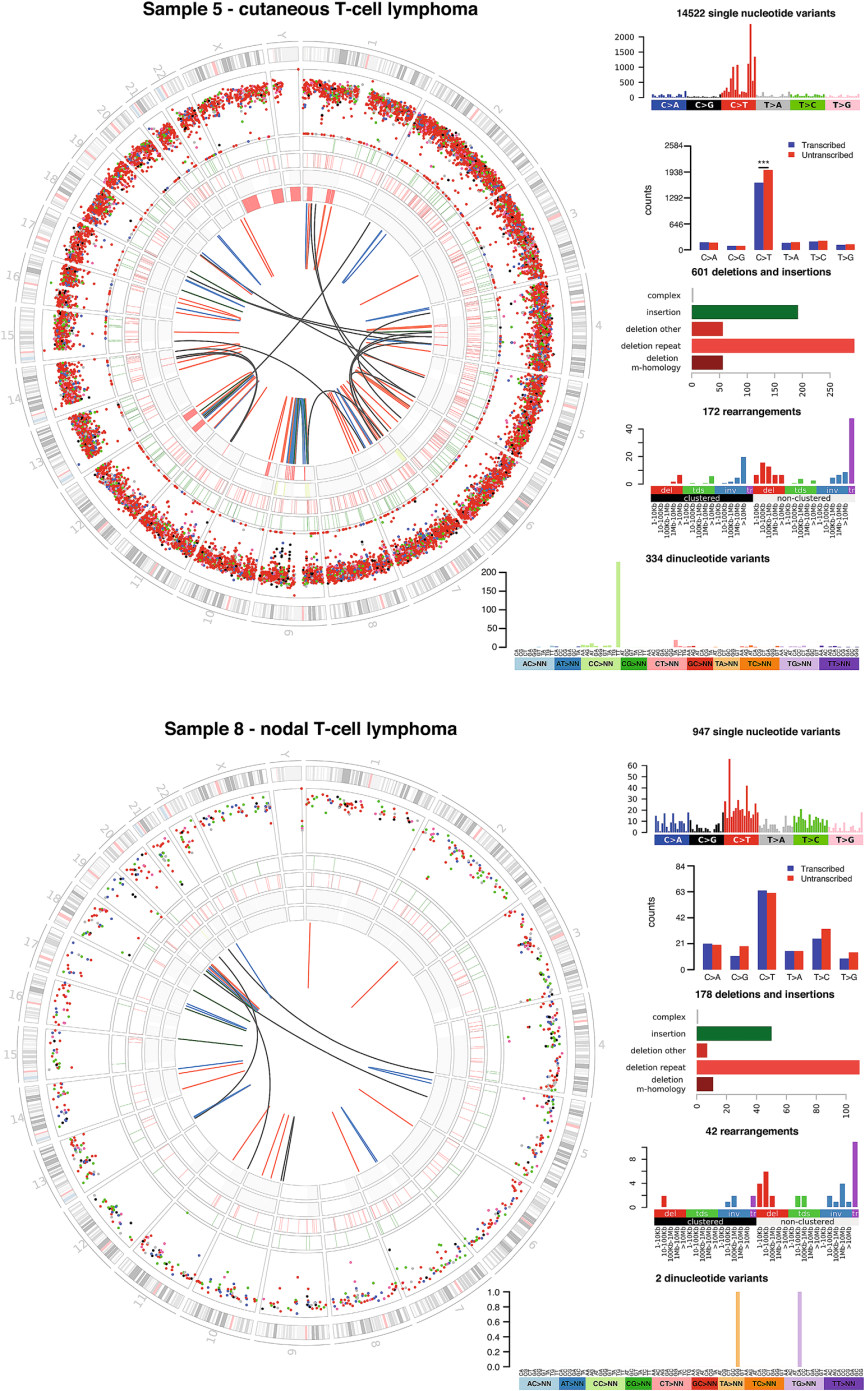
Characteristic features of UV induced mutation are observed only in patients with cutaneous T-cell lymphoma. Whole genome sequencing data from the UK 100,000 genomes project comparing representative cutaneous and nodal T-cell lymphoma patients. The highlighted track on the circos plots is a rainfall diagram of SNVs where distance from the centre is proportional to the logarithm of the distance between adjacent SNVs. Cutaneous T-cell lymphoma patients have a characteristic inner ring of C>T variants due to CC>TT substitutions, which are also clear in the light green bar on the dinucleotide variants plot. Single nucleotide variants occurring within gene bodies were assessed to determine whether they affect the transcriptional or non-transcriptional strand. Significant transcriptional strand bias was observed for C>T mutations in cutaneous T-cell lymphoma but not nodal T-cell lymphoma indicating transcription-coupled nucleotide excision repair, a feature of UV induced mutation.

To validate this finding we examined data generated by the UK 100,000 genomes project. Six newly diagnosed and untreated CTCL samples were included and their genomes were found to have a mutational burden of 3–8.4 somatic coding variants per Mb to which signature 7 contributed between 25 and 61%. Two cases of systemic nodal T-cell lymphoma were also sequenced revealing mutational burdens of 0.3 and 3.5 somatic coding variants per Mb with neither showing any contribution from signature 7. Notably, significant bias towards the untranscribed strand was seen for C>T mutations in cutaneous but not nodal T-cell lymphomas with frequent double nucleotide substitutions CC>TT (Fig. 5) both of which are characteristic features of UV induced mutation.

A significant contribution was noted in ATLL from signature 17. Although the aetiology of signature 17 is unknown, this signature is associated with a high mutational load and neoantigen burden subtype of oesophogeal adenocarcinoma^30^ and is enriched in metastatic tissue of colorectal cancer^31^. ATLL patients have a variable course consisting of indolent (smouldering and chronic) and aggressive (lymphoma and acute) subtypes. Hence, we examined whether there was an association between aggressive subtypes and signature 17. Only 1/13 (8%) indolent ATLL exhibited signature 17, whilst 9/32 (28%) aggressive ATLL exhibited signature 17 but this difference did not reach significance in a Fisher’s exact test.

### Significant associations between mutational signatures and driver mutations

**Figure 6:**
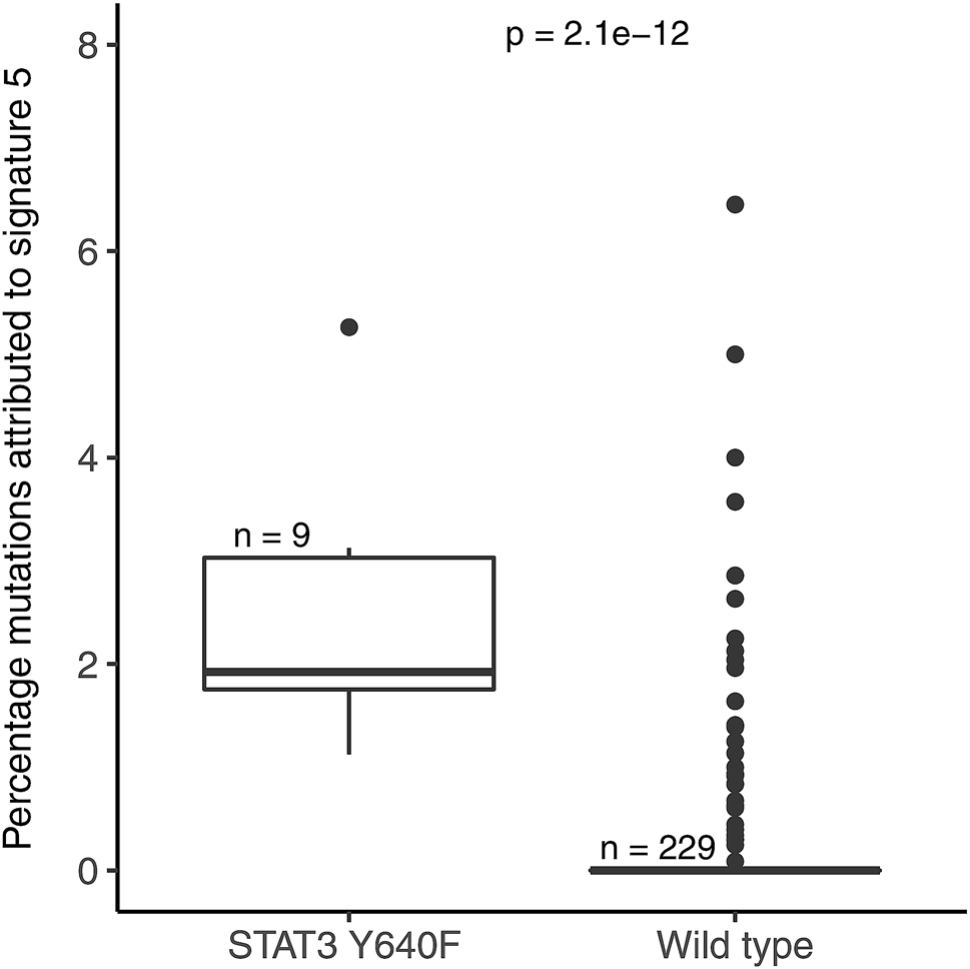
Patients with the STAT3 Y640F mutation showed significantly greater contribution of signature 5 to their mutational catalogue. Wilcoxon rank sum test was used to compare the contribution of each signature between the groups mutant or WT for a given mutation.

**Figure 7:**
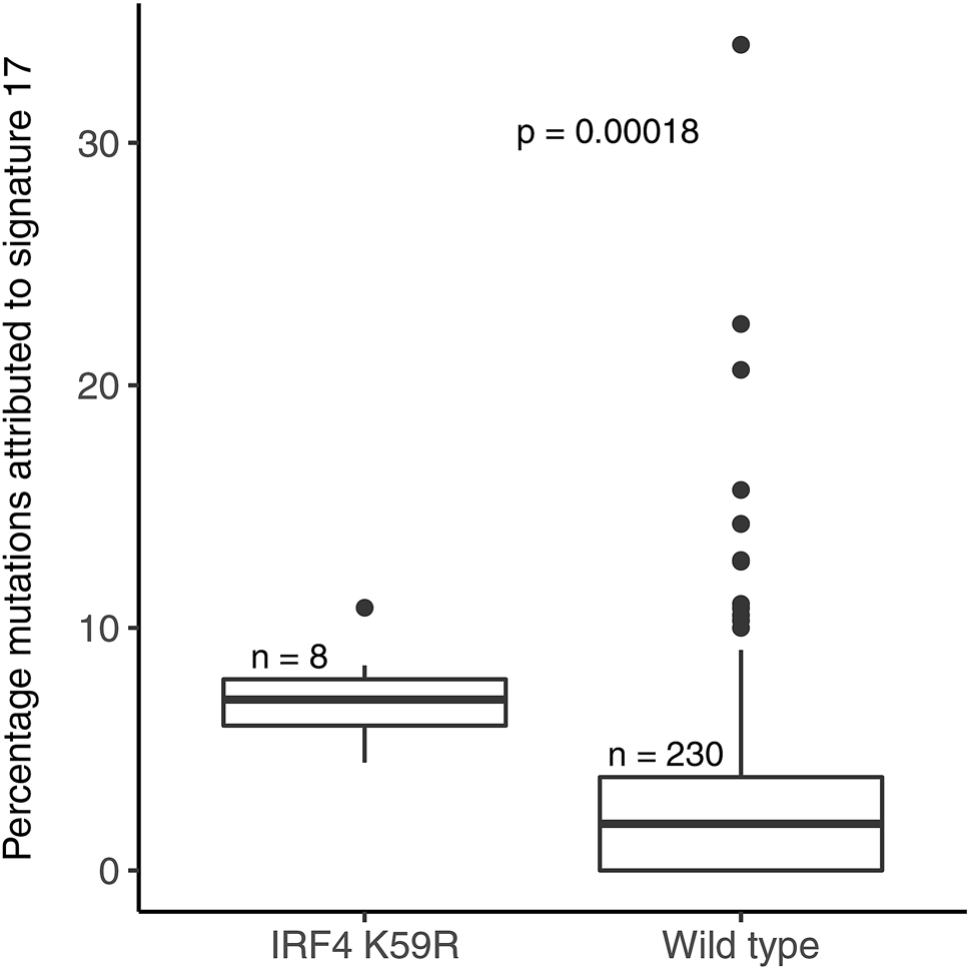
Patients with the IRF4 K59R mutation showed significantly greater contribution of signature 17 to their mutational catalogue. Wilcoxon rank sum test was used to compare the contribution of each signature between the groups mutant or WT for a given mutation.

We used the method of Temko et. al.^32^ to assess the potential causative role of mutational signatures in relation to recurrent driver gene mutations. Causal peaks were used to estimate the contribution of each signature and compared between wild type and mutant groups for each recurrent driver gene mutation which occurred in five or more patients. Using the Bonferroni corrected p-value of 0.00029, two significant associations were observed, between STAT3 Y640F and signature 5 (Fig. 6) and between IRF4 K59R and signature 17 (Fig. 7). Whilst the STAT3 Y640F mutation was distributed across T-cell NHL cases, specifically 7 ATLL, 1 NK/T-cell lymphoma (NKTCL) and 1 MF, the IRF4 K59R mutation was only seen in ATLL patients, consistent with the restriction of signature 17 to these patients. For both of these associations the underlying nucleotide change was most likely to have been generated by the signature observed. No further associations were identified by examining the data on a disease subtype level.

Due to the dominant contributions of C>T peaks in signatures 1, 7 and 30, the only context in which we could clearly define a causal association for signature 7 was for C>T in a TCC context. Of the recurrent driver mutations, only PLCG1 S345F occurs in this context and there was no significant association between this recurrent mutation and signature 7. Additionally this mutation was not restricted to cutaneous lymphoma, being observed in 3 SS, 3 ATLL and 1 AITL patients suggesting it originated from another mutational process. This method of analysis has a much greater power over the whole cohort and within individual disease subtypes with highly recurrent mutations, and so the lack of significant association of specific gene variants with signature 7 causal peaks likely reflects the absence of highly recurrent mutations in CTCL.

### UV mutational signatures in healthy T-cells

If the presence of the UV signature in CTCL reflects prior exposure of the originating T-cell to UV whilst circulating in the skin, we might expect a proportion of healthy peripheral blood memory T-cells to show similar UV induced mutations. In the absence of a clonal proliferation, this can only be identified by looking at the mutational spectrum of single cells. No single cell whole genome or exome sequencing has been performed on healthy T-cells but we did identify a dataset of RNA-seq from CD4 + T-cells where the full length transcript has been sequenced which allows mutation calling^33^. We analysed three subjects where both T_EM_ and T_CM_ single cells had been sequenced. Data processing and mutation calling were performed as described in the supplementary materials. No clear UV signature was observed in 94 cells from Donor 3, 91 cells from Donor 6 or 90 cells from Donor 12, and in each donor a similar mutational burden was observed between T_EM_ and T_CM_ cells. It is estimated that almost twice as many T-cells are permanently resident in the skin than circulating in the blood^34^ but the proportion of T-cells which freely re-circulate through skin and blood is not well characterised. Hence we may need to analyse a much greater number of cells to observe those exposed to UV.

## Discussion

There are several findings in this study which provide insight into the pathogenesis of mature T-cell NHL and have immunobiologic and therapeutic relevance. Strikingly cutaneous T-cell lymphomas, specifically SS and MF variants, show an obvious clonotypic UV signature 7 associated with a high tumour mutational burden as expected for malignancies linked to a specific mutagen. This UV signature contributed 23% and 52% of the mutational load for the SS and MF cohorts respectively. Specifically the UV signature was identified in peripheral blood T-cells from 74% (67/90) of SS patients representing between 7.5 and 88% of the overall SNV mutational burden. Not surprisingly signature 1, associated with age related deamination of methylated cytosines, was detected in all T-cell NHL with the exception of MF in which signature 1 is likely masked by the large contribution of the UV signature. Two mutational signatures were associated with the HTLV-1 associated ATLL cohort namely signature 1, and signature 17 which is of unknown cause but associated with gastric cancer^35^ and oesophageal adenocarcinoma^30^. Our computational power to deconvolute mutational signatures reflects the total number of tumours compared to the mutational load^36^ and therefore the smaller sample size and low mutational burden of the other T-cell NHL cohorts limits an accurate interpretation. Future whole genome studies, particularly of non-cutaneous T-cell NHL subtypes, could yield additional insight into mutational processes as greater coverage would allow increased power for signature extraction.

MF is derived from skin resident effector memory T-cells and, although previous studies suggested that leukaemic T-cells from SS patients were derived from central memory T-cells^37^, the presence of a UV signature in DNA derived from peripheral blood CD4 + T-cells of SS patients supports data that T_CM_ exhibit marked plasticity contributing to circulation from skin through peripheral blood^38^. Indeed, studies have shown that low dose solar simulated irradiation of whole skin causes alterations of gene expression in peripheral blood derived T-cells^39^ suggesting that UVR may have a direct effect on the blood transcriptome of T-cells circulating through the skin. Our previous study of MF suggested that p53 gene mutations were characteristic of UV damage^40^ and several whole exome/genome datasets including our own have suggested a possible UV influence^23,24,41^. Importantly, exposure of cultured cells to simulated solar radiation followed by sequencing of clones generates a mutational profile which replicates that of signature 7^42^ providing molecular evidence for the link between UV and signature 7. Combining data from multiple whole exome T-cell NHL studies has allowed us to confirm that this UV signature is associated with and restricted to CTCL.

Overall 41% of SS patients were treatment naïve and a UV signature was detected in a high proportion of these patients. Other patients received various treatments including photopheresis, in which an enriched population of white blood cells is exposed to psoralens and UVA extra-corporeally and reinfused, and PUVA phototherapy consisting of oral psoralens and UVA irradiation of skin. However the mutational signatures detected are based on the analysis of of clonotypic causal peaks. Therefore critically our findings reflect the impact of environmental UV on the original skin resident T-cell prior to clonal expansion rather than subclonal evolution of the malignant T-cell as subsequent UV induced mutations would not be present in all tumour cells and therefore would not be detectable as a clonotypic signature. Furthermore PUVA phototherapy and photopheresis are characterised mostly by UVA induced cross linking of DNA by psoralens to produce pyrimidine mutations in a TpA sequence context. Until recently mutational signatures have been based on single base substitutions, but analysis of large whole genome cancer datasets has now expanded the number of distinct signatures after identification of those associated with double base substitutions and indels^43^. Specifically this study has decomposed the UV signature into four constituent types with two consisting of C>T at TCN and C>T at CCN, whilst the other two consist of T>A at NTT and T>C at NTT sites. In fact our analysis of T-cell NHL cases submitted to the UK national 100,000 WGS project has confirmed a high frequency of CC>TT double substitutions at diprimidine sites with very high mutational burdens confined only to untreated CTCL cases. In addition there was a significant bias towards C>T mutations in the unstranscribed strand in the CTCL cases.

Epidemiological case–control studies have suggested that occupational solar UV exposure is a risk factor for NHL in Caucasian populations^44^ however most of the studies in this meta-analysis analysed NHL as a cohort and did not divide according to specific lymphoma subtypes. One European case–control study observed a link between MF and occupational sun exposure^45^. In contrast population based registry data from Australia suggested that the incidence of NHL, including cutaneous lymphomas, shows an inverse relationship to latitude, suggesting that UV is protective possibly due to immuno-modulatory effects of UV^46^. A study using US SEER data found no overall trend between UV radiation and cutaneous lymphoma risk but did observe that ethnicity significantly modified the association of UVR and risk^47^. This complex picture highlights the challenge of unravelling the role of UV in NHL. Our data provide molecular evidence for a role of UV in the development of cutaneous T-cell lymphoma only, highlighting the need to examine epidemiological data on a subtype specific basis. Assessment of UV exposure is also challenging with larger population based studies relying on location based estimation whilst personal exposure estimation based on questionnaires increases accuracy but limits the study size.

UV radiation is known to have an immune modulatory effect on the skin by inducing a T_reg_ response^48^. What has not been clear is the mutational impact of UV upon skin-resident T-cells. In contrast to other rapidly dividing cells, studies have shown that irradiation of memory CD8 + T-cells ex vivo rapidly induces all DNA damage response pathways and cell cycle checkpoints during the secondary expansion phase and in the absence of repair, CD8 + T-cells are driven to compulsive suicide to prevent transformation^49^. DNA damage repair pathways may therefore play a critical role in preventing UV induced transformation of skin-resident T-cells. Indeed, heterozygote p53+/− mice develop B-cell lymphomas after UV irradiation^50^. Functional studies are now required to investigate the role of UV in the pathogenesis of T-cell lymphomas.

We identified 20 genes harbouring 34 recurrent variants across different subtypes of T-cell lymphoma, the majority of which are restricted to malignancies of haematopoietic and lymphoid origin and influence T-cell signalling pathways. Our statistical analysis of putative driver gene mutations and specific causal peaks within each signature revealed several associations. The recurrent STAT3 Y640F mutation is found in different T-cell NHL including both CTCL and ATLL and confers constitutive STAT3 phosphorylation and increased transcriptional activity^51^. The association of the STAT3 Y604F variant with signature 5 is not surprising as this signature is considered to be a feature of ageing^52^. We were unable to assess the signature association of recurrent STAT5B N642H, detected almost exclusively in EATL/HSTL. This is due to the low mutational load in these subtypes which prevented the assignment of signature contributions on an individual basis. Whole genome sequencing of these subtypes would provide a clearer picture of the mutational processes involved.

The recurrent IRF4 K59R mutation in ATLL was associated with signature 17 unique to ATLL. Mutations of IRF4 are frequent in B-cell malignancies including myeloma^53^, DLBCL^54^ and primary mediastinal large B-cell lymphoma^55^ and IRF4 gene rearrangements are also found in myeloma^56^ and PTCL^57^, but intriguingly the most common point mutation, the K59R variant, is exclusively associated with ATLL^58^. The HTLV-1 Tax gene induces activation of the NF-kB pathway in ATLL and subsequent induction of IRF4, a key transcription factor downstream of NF-kB^59^. Tax gene expression is subsequently downregulated^60^ and transformed T-cells in ATLL accumulate activating mutations of genes involved in TCR, NF-kB, and CD28 signalling pathways including the IRF4 K59R mutant^11^. Functional studies have confirmed that the activating K59R mutation is a key driver in ATLL and its effects include repression of apoptosis and DNA repair^58^. Specifically constitutive expression of IRF4 in T-cells directly reduces expression of critical DNA repair and checkpoint genes and increases the sensitivity of T-cells to genotoxic stress^61^. Therefore the association of causal signature 17 peaks with the IRF4 K59R mutation in ATLL suggests a strong selection pressure for this mutation which could directly generate an increased mutation rate of other genes due to suppression of DNA repair mechanisms. The underlying cause for signature 17 remains unknown although increasing evidence suggests oxidative damage may contribute^62^. The role of IRF4 in repression of DNA repair mechanisms provides further insight into the aetiology of signature 17 in ATLL.

In conclusion the identification of a clonotypic UV mutational signature in MF and CD4 + peripheral blood T-cells from SS patients confirms that environmental UV exposure contributes to the mutational burden and is a likely causal factor in the transformation of T-cells which are either circulating through or resident in the skin. Importantly these findings also raise the possibility that non-malignant T-cells could acquire UV induced mutations which might modulate normal immune responses.

## Methods

### Data assembly and pre-processing

On 1^st^ June 2017 Pubmed was searched using the terms ("Next Generation Sequencing"[All Fields] OR "Whole Exome Sequencing"[All Fields]) AND ("T cell lymphoma"[Tiab] OR "Mycosis Fungoides"[Tiab] OR "Sezary Syndrome"[Tiab] OR "T cell prolymphocytic leukaemia"[Tiab] OR "T cell large granular lymphocyte leukaemia"[Tiab] OR "Adult T cell leukemia/lymphoma"[Tiab] OR "CD30 positive T cell lymphoproliferative disorder"[Tiab]) NOT ("review"[Publication Type]) and Scopus was searched using the terms ((ALL ("Next Generation Sequencing") OR ALL ("Whole Exome Sequencing")) AND (TITLE-ABS-KEY ("T cell lymphoma") OR TITLE-ABS-KEY ("Mycosis Fungoides") OR TITLE-ABS-KEY ("Sezary Syndrome") OR TITLE-ABS-KEY ("T cell prolymphocytic leukaemia") OR TITLE-ABS-KEY ("T cell large granular lymphocyte leukaemia") OR TITLE-ABS-KEY ("Adult T cell leukemia/lymphoma") OR TITLE-ABS-KEY ("CD30 positive T cell lymphoproliferative disorder"))) AND (LIMIT-TO (DOCTYPE , "ar") OR LIMIT-TO (DOCTYPE , "le")). The outputs were combined to identify 121 unique reports which were manually curated to identify those which reported whole exome sequencing. Searches were also performed of sequencing archives including NCBI Sequence Read Archive, NCBI dbGap, EMBL European Genome-phenome Archive and EMBL European Nucleotide Archive to check for data which was not covered by the literature search.

To avoid skewing the analysis, we included only studies where full mutation details were included in the published data and studies were excluded if they only listed driver mutations, or genes which were mutated in more than one patient. We also only included samples where mutation calling was performed by comparison to a paired germline control.

Choi *et. al.* only included a list of mutations in potential driver genes so their full mutation list was extracted from a data resource covering all the sequencing of CTCL^63^. Mutations from whole exome data of 403 patients were included and a summary by study and disease subtype is provided in Table 1. Experimental details, including capture and variant calling methods, of each study are included in Supplementary Table 2.

Mutation lists were extracted from the supplementary tables of studies and combined in R^64^. Where the reference assembly was not hg19, co-ordinates were lifted over using the rtracklayer^65^ package. Finally, co-ordinates were sense checked using the GenomicRanges^66^ and BSgenome.Hsapiens.UCSC.hg19^67^ packages to ensure that the listed reference base corresponded to the given location. The full mutation dataset generated and analysed during the study are available in the Dryad repository, https://doi.org/10.5061/dryad.8cz8w9gkm.

Clinical data, including age, sex, disease stage and treatment regime at sampling or prior to sample collection, were collected from each publication where provided but this information was incomplete and resultant clinical groupings were too small for subgroup analyses. Treatment data was available for 50% of the cohort of which 70% were treatment naïve at the time of sampling (Supplementary Table 3).

### Identification of mutational signatures

Combined SNV catalogues, i.e. the count of each mutation type (e.g. C>T) in different trinucleotide contexts (e.g. A[C>T]A), were calculated for each disease subtype and mutational signatures identified using a non-negative least squares fit against the set of 30 COSMIC signatures adjusted for exome trinucleotide frequencies. As exome data is prone to false positives, a post processing algorithm was applied to each subtype to remove signatures which had a minimal impact upon the cosine similarity between the SNV catalogue and that reconstructed using the identified signature contributions (maximum allowed reduction in cosine similarity was 0.05).

For identification of signatures on an individual patient level, fitting was restricted to the subset of signatures identified on the disease level^29^. Additionally, signatures were removed if assigned to less than 20 mutations in a sample and no signatures were assigned if the cosine similarity between the model and the data was less than 0.6.

### Association between recurrent mutations and signatures

A study by Park et. al^10^ defined 55 driver genes in CTCL using multiple independent analytical pipelines and confirmed their relevance to the wider subset of mature T-cell NHL and so were used in this analysis. SNV catalogues were limited to those specific variants which recurred within these 55 defined driver genes. 34 recurrent variants were identified in three or more patients, however we determined that recurrence in at least five patients was required to identify signature associations as otherwise the power of the statistical test was decreased. A separate analysis of each tumour type was performed but this approach was limited with the exception of ATLL because of the requirement for both a highly recurrent variant and sufficient SNVs per patient to estimate a signature contribution. Causal peaks for each signature were assigned by comparing the profiles of the nine signatures and identifying mutation contexts strongly associated with a particular signature. Summation of the causal peaks for each signature was then used to calculate a percentage contribution of that signature in all patients with more than 20 SNVs. For each recurrent mutation and signature pair, a Wilcoxon rank sum test was used to determine if there was a significant difference in signature contribution between the wild type and mutant groups.

### Validation in UK 100,000 genomes cohort

All samples were processed by Genomics England and analysed through their cancer pipeline. Briefly, samples were prepared using an Illumina TruSeq DNA PCR-Free preparation kit and then sequenced on a HiSeq X generating 150 bp paired-end reads. Quantity and quality of sequence and presence of cross-contamination were assessed for each sample prior to variant calling. Reads were aligned to human reference genome GRCh38-Decoy + EBV by ISAAC then Strelka was used to call small variants and perform tumour-normal subtraction. Gene annotations were used to determine whether mutations within genes affected the transcriptional or non-transcriptional strand.

## Supplementary Information

Supplementary Information 1

Supplementary Information 2

## Data Availability

The dataset generated and analysed during the current study are available in the Dryad repository, https://doi.org/10.5061/dryad.8cz8w9gkm.
