# Supplementary material for: Spectrum of mutational signatures in T-cell lymphoma reveals a key role for UV radiation in cutaneous T-cell lymphoma
Source: Sci Rep. 2021 Feb 17;11:3962. doi: 10.1038/s41598-021-83352-4 (PMC7889847; doi:10.1038/s41598-021-83352-4)
Supplement: Supplementary file 2 — Supplementary Information 2 [file 41598_2021_83352_MOESM2_ESM.pdf]

Spectrum of mutational signatures in T-cell lymphoma reveals a key role for UV radiation in cutaneous T-cell lymphoma

Christine L Jones[1], Andrea Degasperi[2,3], Vieri Grandi[1], Tauanne D Amarante[2,3], Genomics England Research Consortium[4], Tracey J Mitchell[1], Serena Nik-Zainal[2,3] and Sean J Whittaker[1*]

[1] St. John's Institute of Dermatology, School of Basic and Medical Biosciences, King's College London, Guy's Hospital, London, SE1 9RT, United Kingdom.

[2] MRC Cancer Unit, University of Cambridge, Hutchison/MRC Research Centre, Box 197, Cambridge Biomedical Campus, Cambridge, United Kingdom CB2 0XZ

[3] Academic Laboratory of Medical Genetics, Box 238, Lv 6 Addenbrooke's Treatment Centre, Addenbrooke's Hospital, Cambridge, CB2 0QQ

[4] Full list of Genomics England Research Consortium members provided in manuscript.

Supplementary Methods

Mutation calling from single cell RNA-seq data

Data processing was performed as outlined in the GATK best practices for calling variants in RNA-seq. SRA toolkit (http://ncbi.github.io/sra-tools/) was used to fetch fastq files for all T_{EM} and T_{CM} cells from donor 6, 12 and 16. 2-pass STAR[1] alignment was performed using the hg38 assembly and GENCODE v29 known genes annotation to build the genome index. Picard tools (http://broadinstitute.github.io/picard/) was then used to add read group information, sort, mark duplicates and index the BAM file. GATK[2] Split'n'trim was used to remove intronic overhangs followed by base recalibration. Finally, GATK

HaplotypeCaller was used to identify potential variants followed by GATK VariantFiltration to flag variants in a SNP cluster (-window 35 -cluster 3) and those with a Fisher strand value greater than 30 or quality by depth value of less than 2.

The VariantAnnotation[3] package was used to import vcf files into R[4] for further manipulation. Variants were restricted to SNVs which passed all filters applied by VariantFiltration. Variants were further restricted to those with a read depth of at least 6 as this has been shown to give a true positive rate of 68% for variant calling from RNA-seq data[5]. To exclude germline mutations, we excluded any variant which occurred in three or more cells from the same patient. Since the coverage of some genes was rather sparse, we included variants in the final analysis only if the reference allele was correctly called in at least 9 other cells from that patient. Finally, the mutation context of each variant was annotated using the SomaticSignatures[6] package and mutational profiles were examined for presence of the UV Signature 7 by visual inspection looking for a prevalent C>T in a TCC context peak.

Supplementary Tables

Data in Supplementary1_2020_06_24.xls
Supplementary Table 1: Number of mutations contributed by each signature to an individual's total exome SNV count. ND = Signature not included in the analysis as it was not present in the subtype analysis. NSA = No signatures assigned, either because there were too few mutations to clearly assign signatures or because the cosine similarity between model and data was poor (<0.6).

	Disease	Tumour cell selection	Germline cell selection	Exon capture	Average coverage/depth	Variant caller	Variant calling criteria

Choi 2015	SS	CD3+TCRVβ+CD14−CD8−CD19− from peripheral blood if clonal TCRVB detected, otherwise CD3+CD26−CD14−CD8 − CD19−	CD14+CD3−CD8−CD19− monocytes	NimbleGen 2.1 Exome reagent	All >140x Median 220x/184x (tumour/normal)	Not specified	P-value significance threshold determined independently for each tumour
da Silva Almeida 2015	MF/SS	Skin biopsies and/or CD4+ T-cells from peripheral blood with TCR clonal rearrangement	buccal swabs or granulocytes	SureSelect Human All Exon 50 MB kit	143.51× with 95.3% of the target sequence covered by >30×	SAVI	≥15% of tumour reads and absent in germline sample
Jiang 2015	NKTCL	Frozen tumour tissue	peripheral blood	SureSelect Human All Exon 50 MB kit	134.9× with 90.9% of the target sequence covered ≥10×	GATK Unified Genotyper and muTect	Not specified, excluded those reported in matched control, those with low depth, and those described in dbSNP135
Kataoka 2015	ATLL	Peripheral blood, lymph node or other tumour tissue	buccal swabs	SureSelect Human All Exon v4 or v5 kits	113x in tumour, 108x in matched normal	In-house pipeline	(i) Fisher's exact P ≤ 0.01; (ii) ≥5 variant reads in tumor samples; (iii) VAF in tumor samples ≥0.07; and (iv) a VAF in matched normal samples <0.07
McKinney 2017	HSTL	FFPE tumour tissues	unaffected bone marrow	SureSelect Human All Exon 50MB kit	80x	MuTect version 1.1.7	tumor LOD score cutoff 4.5
Moffitt 2017	EATL	FFPE tumour tissues	unaffected bone marrow	SureSelect Human All Exon 50 MB kit	70x	MuTect version 1.1.7	tumor LOD score cutoff 4.5
Palomero 2014	AITL/EATL/NKTCL/PTCL	Tumour biopsy material	blood, buccal swab or non-tumor infiltrated biopsy material	SureSelect Human All Exon 50 MB kit	45× with 84% of the target sequence covered ≥10×	SAVI	>15% of tumour reads and <3% in germline sample
Prasad 2016	SS	CD4+ T-cells from peripheral blood with TCR clonal rearrangement	granulocytes	Nextera rapid capture exome (37 Mb)	37x	MuTect	MuTect standard recommendation

Roberti 2016	EATL	Microdissected FFPE intestinal tissue - area with highest tumour cell content	Microdissected FFPE intestinal tissue -area devoid of neoplastic cells	SureSelect	195x/213x (tumour/normal) ~ 89% of target sequence covered ≥50x	samtools mpileup (v1.2) and VarScan (v2.3.7)	>22% of tumour reads and < 15% of normal with difference between tumour and normal <22%
Sakata-Yanagimoto 2014	AITL/PTCL	Tumour biopsy material	buccal mucosa, bone marrow MNCs without apparent lymphoma infiltration or peripheral blood cells	SureSelect Human All Exon 50Mb or V4 kit	86.5% was analyzed by ≥20 independent reads on average	samtools	P < 0.01, observed in bidirectional reads and allele frequency < 0.1 in the germline
Ungewickell 2015	MF/SS	Skin biopsies and/or CD4+ clonal Vbeta sorted T-cells from peripheral blood	CD4-/Vbeta- PBMCs	EZexome V2.0 Early Access for Capture by Ambry Genetics	Not specified	GATK SeqGene and VarScan	Default parameters, VAF > 0.1 and supported by bidirectional reads.
Wang 2015	SS	CD4+ T-cells from peripheral blood with TCR clonal rearrangement	fibroblasts	HGSC VCRome 2.1	100× with ~94% of the target sequence covered ≥20	Atlas-SNP2	tumour VAF ≥ 0.04; normal VAF: tumour VAF ≤ 0.15
Woollard 2016	SS	CD4+ T-cells from peripheral blood with TCR clonal rearrangement	fibroblasts	Not specified	82% of the target region covered >20x	VarScan	Varscan default parameters, VAF > 6%
Yoo 2014	AITL	Frozen lymph node tumour biopsy	Buffy coat	SureSelect Human All Exon V3 kit	92.5x	MuTect 1.1.4 and Strelka 1.0.7	(i) ≤1 read with the alternate allele in normal (ii) tumour VAF:normal VAF > 5 (iii) ≤2 reads for the third allele

Supplementary Table 2: Key experimental and bioinformatic parameters of the studies included in this meta-analysis.

	Disease	Number of patients	Percentage treatment naïve at time of sampling
Choi 2015	SS	33	8%
da Silva Almeida 2015	MF/SS	34	Not specified
Jiang 2015	NKTCL	25	100%
Kataoka 2015	ATLL	81	98%
McKinney 2017	HSTL	64	Not specified
Moffitt 2017	EATL	58	Not specified
Palomero 2014	AITL/EATL/ NKTCL/PTCL	12	Not specified
Prasad 2016	SS	12	8%
Roberti 2016	EATL	15	Not specified
Sakata-Yanagimoto 2014	AITL/PTCL	6	Not specified
Ungewickell 2015	MF/SS	11	Not specified
Wang 2015	SS	37	65%
Woollard 2016	SS	10	100%
Yoo 2014	AITL	5	0%

Supplementary Table 3: Treatment status of patients included in this meta-analysis.

Supplementary References

1. Dobin, A. *et al.* STAR: ultrafast universal RNA-seq aligner. *Bioinforma. Oxf. Engl.* **29**, 15–21 (2013).

2. McKenna, A. *et al.* The Genome Analysis Toolkit: A MapReduce framework for analyzing next-generation DNA sequencing data. *Genome Res.* **20**, 1297–1303 (2010).

3. Obenchain, V. *et al.* VariantAnnotation : a Bioconductor package for exploration and annotation of genetic variants. *Bioinformatics* **30**, 2076–2078 (2014).

4. R Core Team (2017). *R: A Language and Environment for Statistical Computing.* (R Foundation for Statistical Computing, Vienna, Austria. URL https://www.R-project.org/).

5. Poirion, O., Zhu, X., Ching, T. & Garmire, L. X. Using single nucleotide variations in single-cell RNA-seq to identify subpopulations and genotype-phenotype linkage. *Nat. Commun.* **9**, 4892 (2018).

6. Gehring, J. S., Fischer, B., Lawrence, M. & Huber, W. SomaticSignatures: inferring mutational signatures from single-nucleotide variants. *Bioinforma. Oxf. Engl.* **31**, 3673–3675 (2015).
